# Health literacy and e-health literacy among Arabic-speaking migrants in Sweden: a cross-sectional study

**DOI:** 10.1186/s12889-021-12187-5

**Published:** 2021-11-25

**Authors:** Lina Bergman, Ulrica Nilsson, Karuna Dahlberg, Maria Jaensson, Josefin Wångdahl

**Affiliations:** 1 Division of Nursing, Department of Neurobiology, Care Sciences and Society, Karolinska Institute, Box 23 300, 141 83 Huddinge, Sweden; 2 Department of Perioperative Medicine and Intensive Care, Karolinska University Hospital, Stockholm, Sweden; 3 School of Health Sciences, Faculty of Medicine and Health, Örebro University, Örebro, Sweden; 4 Department of Public Health and Caring Sciences, Uppsala University, Uppsala, Sweden

**Keywords:** E-health literacy, eHEALS, Ethnic minorities, Migrant health, Health literacy, HLS-EU-Q16

## Abstract

**Background:**

Health inequities arise when the public cannot access and understand health information in an easy, accessible, and understandable way. Evidence supports that health literacy (HL) is a determinant for health outcomes, and when HL is limited this may have a major impact on morbidity as well as mortality. Migrants are known to have limited HL. Therefore, this study aimed to explore comprehensive health literacy (CHL) and electronic health literacy (eHL) among Arabic-speaking migrants in Sweden.

**Methods:**

This was a cross-sectional observational study conducted in Sweden. A total of 703 persons were invited to participate between February and September 2019. Two questionnaires – the Health Literacy Survey European Questionnaire (HLS-EU-Q16) and the eHealth Literacy Scale (eHEALS) – and questions about self-perceived health and Internet use were distributed in Swedish and Arabic. Various statistical analyses were performed to determine the associations for limited CHL and eHL.

**Results:**

A total of 681 respondents were included in the analysis. Of these, 334 (49%) were native Arabic-speaking migrants and 347 (51%) were native Swedish-speaking residents. CHL and eHL differed between the groups. The Arabic speakers had significantly lower mean sum scores in eHL 28.1 (SD 6.1) vs 29.3 (6.2), *p* = 0.012 and lower proportion of sufficient CHL 125 (38.9%) vs 239 (71.3%), *p* < 0.001 compared to Swedish speakers. Multiple regression analysis showed on associations between limited CHL and eHL and being Arabic speaking, less Internet use, and not finding the Internet to be important or useful. Furthermore, longer time spent in Sweden was associated with higher levels of CHL among the Arabic speakers, (OR 0.94, 95% CI 0.91–0.98, *p* < 0.01).

**Conclusions:**

CHL and eHL differ between Arabic-speaking migrants and native Swedish speakers, but also between Arabic speakers who have lived different lengths of time in Sweden. Though it seems that the eHealth literacy is less affected by language spoken, the Internet is suggested to be an appropriate channel for disseminating health information to Arabic-speaking migrants.

**Supplementary Information:**

The online version contains supplementary material available at 10.1186/s12889-021-12187-5.

## Background

Good health comprises of physical, mental, and social well-being. In order to achieve this, the person need to have sufficient, reasonable, and affordable access to healthcare regardless of their gender, race, social position, or ethnicity. However, there are today inequalities in the health status of different social groups, and evidence shows that social factors such as socio-economic position, gender, and ethnicity influence the health of individuals [1]. One of the most dominant issue today is international migration. The number of international migrants is estimated to be 272 million (3.5% of the world’s population) and of those about 26 million are refugees [2] i.e., persons fleeing armed conflict or persecution [3]. Migrants mostly includes people who have decided to move to improve their lives by finding work as well as for family reunion, education [3].

Globally, it has been reported that migrants often do not seek health care, supposedly due to a limited knowledge of their rights or to cultural or language barriers [4]. It has also been recognised that for people to use health care services appropriately they need to be able to access and understand health-related information, as captured in the concept of health literacy (HL) [5, 6] In this paper, we refer to the comprehensive definition of HL by Sorensen et al. [7]: *“Health literacy is linked to literacy and entails people’s knowledge, motivation and competences to access, understand, appraise and apply health information in order to make judgements and take decisions in everyday life concerning health care, disease prevention and health promotion to maintain or improve quality of life during the life course”(page 3).* Another more explicit form of HL is electronic health literacy (eHL). According to Norman et al. [8], eHL is” *the ability to seek out, find, evaluate and appraise, integrate, and apply what is gained in electronic environments towards solving a health problem” (page 2)*. Both comprehensive health literacy (CHL) and eHL are important in everyday life. Today, health information has become more advanced, technical, and complex [9, 10] and medical jargon makes the information very difficult to understand, especially in a new language [9]. Moreover, electronic health (eHealth) is increasingly being integrated into the delivery of healthcare services and has been recognised by the World Health Organization as one potential solution to improving health services and moving towards achieving the sustainable development goals [11]. However, recent evidence suggests that eHL is generally overlooked in developing eHealth interventions targeting socially disadvantaged groups, and this might lead to health disparities [12].

Worldwide, one of the most common native languages among migrants and refugees is Arabic [13]. In Sweden, about 200,000 refugees speak Arabic, of which most are Syrians [14]. Importantly, Arabic migrants are not a homogenous group; thus, their health status may vary among individuals. However, previous research has shown that many Arabic refugees have poor self-assessed health and poor mental health [5, 15]. Other common health problems are smoking [6], physical inactivity, obesity, hypertension, and diabetes [16]. Also, recent research findings have shown that migrants in Sweden have higher mortality related to COVID-19 compared to Swedish-born persons [17]. Research further shows that up to 73% of the Arabic-speaking refugees in Sweden refrain from seeking healthcare even if they need it, which is partly due to language problems, to the idea that help will not be given, and to a lack of knowledge about where to go for help [5, 18]. Studies from both Sweden and Turkey report that about 60% of all Arabic-speaking refugees have limited CHL [5, 19]. Associations between limited CHL and poor general self-rated health and impaired mental well-being have been reported [20, 21].

To reduce injustices due to limited HL in the population, it is vital to explore CHL and eHL among subgroups of the population, for example, between those who have and do not have a country’s official language as their native language and between subgroups who have lived in the country for different lengths of time. However, studies on levels of CHL and eHL among Arabic-speaking migrants in comparison with native Swedish-speaking residents in Sweden are lacking. Thus, an understanding of health vulnerabilities as well as resilience factors (i.e., resources needed to cope with health problems or resist the impact of health hazards) is crucial to improving migrants’ health [2].

### Aim

Our aim was to explore CHL and eHL among Arabic-speaking migrants in Sweden by addressing the following research questions:

1. Does CHL/eHL differ between Arabic-speaking migrants and native Swedish speakers?
2. What are the differences and similarities regarding scoring of CHL/eHL items between Arabic-speaking migrants and native Swedish speakers?
3. Is length of stay in Sweden among Arabic-speaking migrants associated with CHL/eHL?

## Methods

### Study design

This was a cross-sectional observational study performed as a part of a larger project that included the translation and validation of two HL questionnaires into Swedish and Arabic [22–24]. This study was approved by the Swedish Ethical Review Authority (Dnr 2019/5:1) and followed the ethical principles of the World Medical Association outlined in the Declaration of Helsinki and subsequent amendments [25]. All participants received written and verbal information about the study design, including information about the voluntary nature of participation and that they could withdraw from the study at any time. They were also guaranteed confidentiality and secure data storage.

### Participants and data collection

Two different language groups in the Swedish population were recruited, and data were collected between February and September 2019. One group included participants who were native speakers of Arabic (hereafter referred to as Arabic speakers), and the other group included participants who were native speakers of Swedish (hereafter referred to as Swedish speakers). Convenience and snowball sampling techniques were applied aiming for a diversity in sex, age, and level of education. The last author (JW) visited a total of nine arenas for recruitment of Arabic speakers and 12 arenas for recruitment of Swedish speakers. Arenas for recruitment included university courses, adult education courses, and civic orientation courses as well as larger workplaces and non-governmental organizations. The different arenas selected for recruitment were visited at one or more time point during the data collection period. Potential participants were informed verbally and in writing about the study design by either the last author (JW) or by key stakeholders (i.e., organisation managers or others) selected by the researcher. The study aimed for a target sample of 300 participants in each group, as informed by the recommended sample size for the main purpose of this project., i.e. the psychometric validation studies [26]. Hence, a total of sample of a minimum of 600 participants was considered appropriate. Inclusion criteria were being an adult (≥18 years of age), having sufficient skills to read, understand, and complete the questionnaire in their native language (Swedish or Arabic), and availability on the days for data collection. Participants were asked to complete the questionnaire at the study site. Completed questionnaires were then collected by the researcher or the key stakeholder and return of the questionnaire implied consent to participate.

### Study questionnaires and additional questions

Two questionnaires, the Health Literacy Survey European Questionnaire (HLS-EU-Q16) and the eHealth Literacy Scale (eHEALS), were distributed in Swedish or Arabic according to the participant’s native language. We also collected socio-demographic data (age, biological sex, country of birth, education) and number of years lived in Sweden (only for the Arabic speakers). In addition, one question about health and three questions about Internet use were included. The question about general self-perceived health – *How do you assess your overall health status?* – had the following response options: very poor, poor, fair, good, or very good [27, 28]. Use of the Internet was assessed by the question *How often do you use the Internet*? and had the following response options: almost every day, several days a week, around once a week, or less than 1 day a week [29]. Usability of the Internet was measured with the question *How useful is the Internet in helping you make decisions about your health?* and had the following response options: not useful at all, not very useful, unsure, useful, or very useful. Finally, the importance of the Internet was measured with the question *How important is it for you to be able to access health resources on the Internet?* and had the following response options: not important at all, not very important, unsure, important, or very important [8].

The HLS-EU-Q16 scale [30] is a short version of the HLS-EU-Q47 developed by Sorensen et al. [31]. It has 16 items aiming to assess CHL, that is, the knowledge, motivation, and competence needed to access, understand, appraise, and apply health information. The respondents are asked to rate their perceived difficulty of a given task using a four-point Likert scale (very easy, easy, difficult, or very difficult). In this study, an HLS-EU-Q16 index sum score was calculated ranging from 0 to 16, where higher scores represented higher self-perceived CHL. Moreover, the index sum scores were categorised according to the threshold values of inadequate (0–8 points), problematic (9–12 points), and sufficient (13–16 points) and then further dichotomised into limited (inadequate + problematic = 0–12 points) and sufficient (13–16 points) [30]. The instrument is psychometrically evaluated in several different languages, including Arabic [30, 32–37]. Our research group are performing psychometric evaluations of the Arabic and Swedish versions of the HLS-EU-Q16, and the results indicates satisfactory validity, unpublished manuscripts.

The eHEALS scale was developed by Norman and Skinner in 2006 and aims to measure literacy skills useful in assessing the effects of strategies to deliver online information and applications [8, 38]. The eHEALS scale consists of 8 items, where respondents are asked to rate each item on a five-point Likert scale (strongly disagree, disagree, neither, agree, or strongly agree). Total scores range from 8 to 40 with higher scores indicating higher self-perceived eHL [8]. eHEALS scores were dived into the threshold values of inadequate (8–20 points), problematic (21–26 points), and sufficient (27–40 points), as well as dichotomised into limited (inadequate + problematic = 8–26 points) and sufficient (27–40 points) [22, 23]. Psychometric testing of eHEALS indicates that it is a valid and reliable instrument [8, 38].

, and it has further been translated, adapted, and validated into Swedish and Arabic [22, 23].

### Statistical analysis

Data are presented as frequency, percent, mean, median, standard deviation (SD), inter quartile range, and range. The chi-square test (or Fisher’s exact test) was used to analyse differences in sex; education level; general self-perceived health; the frequency, usability, and importance of the Internet, the distribution of HLS-EU-Q16/eHEALS levels. Student’s t-test was used for analysing differences in age, HLS-EU-Q16 index sum score and eHEALS sum score. The associations between limited CHL and eHL (dependent variables) and independent variables were evaluated by univariate- and multiple logistic regression analysis. Independent variables were native language, biological sex, age, years in Sweden, educational level, general self-perceived health and the three variables measuring internet use. Internet use were included based on previous research showing positive associations between internet use and eHL [22, 23] as well as that better HL are associated with higher degrees of health-related information-seeking behavior online [39]. The multiple regression models were tested using forced entry methods, model fit was assessed using omnibus tests of model fit, and Nagelkerke R squared was used to assess variance. The results are presented as unadjusted (crude) and adjusted odds ratios with 95% confidence interval (CI). For the item analysis, answers for the items on the HLS-EU-Q16 and eHEALS were dichotomized. HLS-EU-Q16, difficult and very difficult were combined, and easy and very easy. eHEALS, disagree and strongly disagree were combined, and neither, agree, strongly agree. Thereafter Chi-square tests were performed to investigate differences in answers between the Arabic- and Swedish speakers. Finally, univariate and multiple logistic regression was performed to examine the association between limited CHL and eHL and length of stay in Sweden in the Arabic speaking group. In addition to the independent variables in the multiple models described above, the length of stay in Sweden was included in these multiple regression models. All data were analysed using IBM SPSS Statistics version 27 (IBM Corp), and a two-tailed *p*-value < 0.05 was considered significant.

## Results

### Characteristics of the study population

A total of 703 persons were invited to participate, of whom 22 declined. Thus, 681 respondents were included in the analysis. Of these, 334 (49%) were Arabic speakers and 347 (51%) were Swedish speakers. In the total sample, the mean age was 45.9 years (SD 18.3 years). There was a higher proportion of females (57%), almost half of the participants had graduated from university (49%), and one-third perceived their general health as good or better (77%). The majority, 55%, had sufficient CHL, and 68% had sufficient eHL.

There was a higher proportion of females (57%), almost half of the participants had graduated from university (49%), and one-third perceived their general health as good or better (77%). The majority, 55%, had sufficient CHL, and 68% had sufficient eHL.

There were significant differences in the distribution of biological sex, age, educational level, general self-perceived health, and perceptions of the usability and importance of the Internet between the groups of Arabic speakers and Swedish speakers. For the Arabic speakers, the most common country of birth was Syria (59%) and the respondents had on average lived 9.6 (SD 8.4) years in Sweden (Table 1).

**Table 1:** Demographics of the study population

Characteristics	Total (*n* = 681)	Arabic speakers (*n* = 334)	Swedish speakers (*n* = 347)	*p* value
Biological sex, n (%)				
Female	382 (56.9)	206 (62.8)	176 (51.2)	0.003^a^
Male	290 (43.1)	122 (37.2)	168 (48.8)	
Age in years				
Mean (SD)	45.9 (18.3)	42.1 (12.7)	49.4 (21.7)	< 0.001^b^
Range	19–95	19–77	19–95	
Country of birth, n (%)				–
Sweden	348 (51.3)	3 (0.9)	345 (99)	
Syria	197 (29.1)	197 (59.2)	–	
Iraq	74 (10.9)	74 (22.2)	–	
Other	59 (8.7)	59 (17.7)	–	
Years in Sweden				–
Mean (SD)	–	9.6 (8.4)	–	
Range	–	0–38	–	
Highest education level, n (%)				
None	6 (0.9)	6 (1.8)	–	< 0.001^a^
1–6 years	31 (4.6)	26 (7.8)	5 (1.5)	
7–9 years	77 (11.4)	49 (14.8)	28 (8.1)	
10–12 years	232 (34.3)	75 (22.6)	157 (45.6)	
Graduated from university	330 (49.8)	176 (53.0)	154 (44.8)	
Generated self-perceived health, n (%)				
Very poor	6 (0.8)	6 (1.8)	–	< 0.001^a^
Poor	27 (4.0)	19 (5.7)	8 (2.3)	
Fair	123 (18.1)	83 (24.9)	40 (11.5)	
Good	354 (52.1)	144 (43.2)	210 (60.5)	
Very good	170 (25.0)	81 (24.4)	89 (25.6)	
Frequency of Internet use, n (%)				
Almost never	20 (2.9)	7 (2.1)	13 (3.7)	0.223^a^
Less than 1 day a week	7 (1.0)	6 (1.8)	1 (0.3)	
Around 1 day a week	18 (2.6)	9 (2.7)	9 (2.6)	
Several days a week	51 (7.5)	27 (8.1)	24 (6.9)	
Almost every day	584 (85.9)	284 (85.3)	300 (86.5)	
Usability of the Internet, n (%)				
Not useful at all	23 (3.4)	8 (2.4)	15 (4.4)	0.017^a^
Not very useful	35 (5.2)	22 (6.7)	13 (3.8)	
Unsure	133 (19.9)	75 (22.7)	58 (17.1)	
Useful	315 (47.1)	138 (41.8)	177 (52.2)	
Very useful	163 (24.4)	87 (26.4)	76 (22.4)	
Importance of the Internet, n (%)				
Not important at all	25 (3.7)	11 (3.3)	14 (4.1)	0.039^a^
Not very important	36 (5.4)	20 (6.1)	16 (4.7)	
Unsure	120 (17.9)	73 (22.1)	47 (13.8)	
Important	278 (41.5)	134 (40.6)	144 (42.4)	
Very important	211 (31.5)	92 (27.9)	119 (35.0)	

^a^Chi-square test (or Fisher’s exact test) ^b^t-test

Abbreviation: *SD* Standard deviation

### Does CHL/eHL differ between Arabic speakers and Swedish speakers?

The proportion of Arabic speakers who had inadequate or problematic CHL was significantly higher compared to Swedish speakers. Arabic speakers had also significantly lower mean sum scores for eHL compared to the Swedish speakers (Table 2).

**Table 2:** Distribution and comparison of HLS-EU-Q16 and eHEALS among Arabic and Swedish speakers

Variables	Total (*n* = 681)	Arabic Speakers (*n* = 334)	Swedish speakers (*n* = 347)	*p*-value
HLS-EU-Q16, n (%)				
Inadequate (0–8)	87 (13.3)	66 (20.6)	21 (6.3)	< 0.001^b^
Problematic (9–12)	205 (31.2)	130 (40.5)	75 (22.4)	
Sufficient (13–16)	364 (55.5)	125 (38.9)	239 (71.3)	
eHEALS sum score				
Mean (SD)	28.7 (6.2)	28.1 (6.1)	29.3 (6.2)	0.012^a^
Range	8–40	8–40	8–40	
eHEALS, n (%)				
Inadequate (8–20)	48 (7.7)	27 (9.0)	21 (6.5)	0.005^b^
Problematic (21–26)	154 (24.8)	89 (29.8)	65 (20.1)	
Sufficient (27–40)	420 (67.5)	183 (61.2)	237 (73.4)	

^a^ t-test ^b^ Chi-square (or Fisher’s exact test)

### Is there any association between being an Arabic speaker and limited CHL/eHL?

The analysis showed that being Arabic speaking was associated with limited CHL, with an odds ratio of 3.9 (95% CI 2.82–5.41, *p* < 0.01) compared to being Swedish speaking. The association remained (OR 3.65; 95% CI 1.95–6.82, *p* < 0.01) after adjusting for demographic factors, self-perceived health, frequency of Internet use, and importance and usability of the Internet to find information about one’s own health. Lower educational level and lower perceptions of the Internet as useful and important were also significantly associated with limited CHL, all other factors being equal (Table 3).

**Table 3:** Logistic regression predicting the probabilities of having limited HL (HLS-EU-Q16) in the Arabic study population

Variables	Crude OR	(95% CI)		Adjusted OR	(95% CI)	
Native language						
Swedish	Ref.			Ref.		
Arabic	3.90**	2.82	5.41	3.65**	1.95	6.82
Biological sex						
Male	Ref.			Ref.		
Female	1.53*	1.11	2.09	1.34	0.93	1.94
Age						
19–24	Ref.			Ref.		
25–54	1.49	0.90	2.47	0.90	0.49	1.65
55–64	1.03	0.54	1.97	0.60	0.28	1.28
65+	0.76	0.41	1.38	0.52	0.25	1.09
Education						
Academic education	Ref.			Ref.		
7–12 years	1.58*	1.15	2.17	1.68*	1.14	2.48
0–6 years	4.20**	1.88	9.37	2.34	0.90	6.05
General self-perceived health						
Very good or good	Ref.			Ref.		
Neither, bad, or very bad	2.91**	2.11	4.01	1.02	0.57	1.83
Frequency of Internet use						
Every or several days perweek	Ref.					
One day per week or less	2.65*	1.34	5.25	1.44	0.62	3.38
Usability of the Internet						
Useful or very useful	Ref.			Ref.		
Unsure	2.63**	1.76	3.93	1.89*	1.15	3.11
Not very useful or not useful at all	3.91**	2.15	7.13	2.55*	1.11	5.87
Importance of the Internet						
Important or very important	Ref.			Ref.		
Unsure	2.87**	1.88	4.39	2.00*	1.15	3.46
Not very important or not important at all	3.27**	1.85	5.78	1.97	0.88	4.40

Ref. = reference category Adjusted OR (full model) R^2^ = 7.66 (Hosmer & Lemeshow), 0.19 (Cox & Snell), 0.25 (Nagelkerke). Model χ^2^ (1) = 131.5, *p* < 0.01. *Significant at *p* < 0.05 **Significant at *p* < 0.01. Abbreviations: *CI* Confidence interval, *OR* Odds ratio

The analysis showed that being Arabic speaking was also associated with limited eHL, with an odds ratio of 1.75 (95% CI 1.24–2.45, *p* < 0.01) compared to being Swedish speaking. The association remained (OR 2.35; 95% CI 1.13–4.86, *p* < 0.05) after adjusting for demographic factors, self-perceived health, frequency of Internet use, and the importance and usability of the Internet to find information about one’s own health. Lower educational level, Internet use of 1 day per week or less, and lower perceptions of the Internet as useful and important were also significantly associated with limited eHL, all other factors being equal (Table 4).

**Table 4:** Logistic regression predicting the probabilities of having limited eHL (eHEALS) in the study population

Variables	Crude OR	(95% CI)		Adjusted OR	(95% CI)	
Native language						
Swedish	Ref.			Ref.		
Arabic	1.75**	1.24	2.45	2.35*	1.13	4.86
Biological sex						
Male	Ref.			Ref.		
Female	1.05	0.75	1.48	0.89	0.58	1.36
Age						
19–24	Ref.			Ref.		
25–54	1.08	0.61	1.91	0.69	0.34	1.41
55–64	1.66	0.82	3.36	1.03	0.44	2.41
65+	2.51*	1.32	4.76	1.65	0.74	3.70
Education						
Academic education	Ref.			Ref.		
7–12 years	1.58*	1.11	2.25	1.68*	1.07	2.63
0–6 years	2.94**	1.42	6.08	1.63	0.63	4.20
General self-perceived health						
Very good or good	Ref.			Ref.		
Neither, bad, or very bad	1.60*	1.14	2.24	1.03	0.53	2.00
Frequency of internet use						
Every or several days per week	Ref.			Ref.		
One day per week or less	11.17**	4.54	27.45	4.03*	1.33	12.24
Usability of the Internet						
Useful or very useful	Ref.			Ref.		
Unsure	5.35**	3.45	8.29	3.04**	1.81	5.09
Not very useful or not useful at all	15.77**	7.82	31.81	6.77**	2.80	16.38
Importance of the Internet						
Important or very important	Ref.			Ref.		
Unsure	6.17**	3.92	9.72	2.56**	1.47	4.46
Not very important or not important at all	8.02**	4.38	14.69	1.74	0.76	4.01

Ref. = reference category Adjusted OR (full model) R^2^ = 0.21 (Hosmer & Lemeshow), 0.23 (Cox & Snell), 0.33 (Nagelkerke). Model χ^2^ (1) = 157.5, *p* < 0.01. *Significant at *p* < 0.05 **Significant at *p* < 0.01. Abbreviations: *CI* Confidence interval, *OR* Odds ratio

### What are the differences and similarities regarding the scoring of CHL/eHL items between Arabic and Swedish speakers?

A higher proportion of Arabic speakers answered difficult or very difficult on 14 of the 16 items on the HLS-EU-Q16 scale compared with the proportion of Swedish speakers. On the item level, the largest difference between the two groups was found for the item “*Find out where to get professional help when you are ill” * (Table 5).

**Table 5:** Proportions of the rated difficulties on the HLS-EU-Q16 items in Arabic speakers (*n* = 334) and Swedish speakers (*n* = 347)

Dimensions and items	Arabic speakers	Swedish speakers	
	Difficult / very difficult^a^ (%)	Difficult / very difficult^a^ (%)	*p* value^b^
**Accessing**			
Find out where to get professional help when you are ill	50.9	13.4	< 0.001
Find information on treatments of illness that concerns you	34.3	10.3	< 0.001
Find information on how to manage mental health problems like stress or depression	47.8	32.5	< 0.001
Find out about activities that are good for your mental health	15.4	10.3	0.049
**Understanding**			
Understand your doctor’s or pharmacist’s instruction on how to take a prescribed medicine	17.4	2.6	< 0.001
Understand health warnings about behaviour such as smoking, low physical activity, and drinking too much	16.0	4.1	< 0.001
Understand why you need health screening	16.9	5.5	< 0.001
Understand what the doctor says to you	30.5	7.6	< 0.001
Understand information in the media on how to get healthier	25.8	16.1	0.002
Understand advice on health from family members or friends	13.0	10.9	0.399
**Appraising**			
Judge which everyday behaviour is related to your health	19.1	6.2	< 0.001
Judge when you may need to get a second opinion from another doctor	58.5	45.0	< 0.001
Judge if the information on health risks in the media is reliable	43.4	32.7	0.004
**Applying**			
Follow instruction from your doctor or pharmacist	14.8	2.1	< 0.001
Use information the doctor gives you to make decisions about illness	37.9	21.0	< 0.001
Decide how you can protect yourself from illness based on information in the media	26.2	28.4	0.532

^a^In the analysis, difficult and very difficult were combined, and easy and very easy. Only difficult/very difficult are presented in the table. ^b^Chi-square test

For the eHEALS scale, a significantly higher proportion of Arabic speakers answered disagree or disagree strongly on three of the eight items compared with the proportion of Swedish speakers. The largest difference was found for the item *“I feel confident in using information from the Internet to make health decision*” (Table 6).

**Table 6:** Proportions of the rated not disagree on the eHEALS items in Arabic speakers (*n* = 334) and Swedish speakers (*n* = 347)

Items^a^	Arabic speakers	Swedish speakers	*p* value^b^
	Disagree / Strongly disagree^a^ (%)	Disagree /Strongly disagree^a^ (%)	
I know how to find helpful health resources on the Internet	14.4	8.4	0.014
I feel confident in using information from the Internet to make health decision	16.3	9.1	0.006
I know where to find helpful health resources on the Internet	9.3	7.4	0.360
I know how to use the Internet to answer my health questions	11.9	8.9	0.206
I know what health resources are available on the Internet	11.5	12.6	0.658
I can tell high quality from low quality health resources on the Internet	17.8	12.1	0.036
I know how to use the health information I find on the Internet to help me	10.9	8.6	0.320
I have the skills I need to evaluate the health resources I find on the Internet	15.3	14.3	0.724

^a^In the analysis, disagree and strongly disagree were combined, and neither, agree, strongly agree. Only disagree/strongly disagree are presented in the table. ^b^Chi-square test

### Is length of stay in Sweden associated with CHL/eHL among Arabic speakers?

The regression analysis showed that shorter length of stay in Sweden was associated with limited CHL, with an odds ratio of 0.9 (95% CI 0.91–0.97, *p* < 0.01). The association remained (OR 0.94; 95% CI 0.91–0.98, *p* < 0.01) after adjusting for demographic factors, self-perceived health, frequency of Internet use, and importance and usability of the Internet to find information about one’s own health. Lower educational level, and lower perceptions of the Internet as useful and important were also significantly associated with limited HL, all other factors being equal (Supplementary Table 1). No significant associations were found between length of stay in Sweden and eHL.

## Discussion

In the present study we found that having Arabic as a native language is strongly associated with limited CHL and eHL even after adjusting for gender, age, and level of education, and this supports previous findings regarding HL among migrants [5, 40–43]. However, a study from the Netherlands found no differences in CHL between most of the ethnic minority groups compared to the indigenous Dutch population, except for the Turkish population that reported significantly lower levels of CHL. The results were adjusted for age, level of education, and gender, and the authors explained these different results between the groups of migrants as perhaps due to the small sample size or to a possible selection bias [44]. However, they had not taken the number of years living in the new country into account, which could perhaps be the reason behind the different results. This was an aspect that we found reduces the chance of limited CHL and eHL in our results. There was an association between shorter time living in Sweden and lower levels of CHL in the Swedish Arabic speaking. It has been reported earlier that over time, as migrants become more familiar with the healthcare system, they improve their HL and thereby use the health information more correctly [45]. It is also known that it takes at least 10 years for a migrant to attain living conditions comparable to the indigenous population of the country [46]. Integration into a new country in all aspects such as learning the language and where to find health information as well as learning to trust the healthcare system takes time. It is known that migrants’ expectations about the new healthcare system are to some extent based on their experiences and knowledge of the healthcare system in their home country [45], and they to a larger extent than the indigenous population do not trust healthcare workers [47]. To expedite and facilitate integration strategies, simultaneous identification with both the host and the home culture should be encouraged [48], as well as providing tailored healthcare information in a plain language [45]. Furthermore, providing assistance in terms of accessing social support and making connections within people’s social environments can facilitate improved health and general wellbeing [49].

In the present study the Arabic speakers rated significant more *Difficult, or very Difficult* in almost all of the HLS-EU-Q16 questions (14 out of 16) compared with the Swedish speakers The greatest difference were found for the item “*Find out where to get professional help when you are ill”*. As discussed above, learning to know and navigate the health care system in a new country as well as language barriers [45] is a possible explanation to this great difference. During the initial period of settling into a new country, migrants’ social networks are usually sparse and support from family and friends in their home country is therefore vital to their health during their first years in the new country [46, 49]. This may explain why there was no significant difference in the item *Understand advice on health from family members or friends*” as well as that only 13% of the Arabic speakers reported this were *difficult or very difficult*. In most cases the migrants develop new contacts within their own group, and a risk factor for poor health is living in a place where for geographical reasons it is difficult to establish contacts with members of one’s own group [46]. Lower eHL levels among the Arabic speakers compared with the Swedish speakers was also seen but only for three out of the eight items. A reason for this “smaller” differences between the groups could be that it is easier for the Arabic speakers to find information on the Internet in their own language compared to finding information in the new country’s media and from healthcare providers. However, when answering the eHEALS we do not know if the Arabic speakers related their answer to information in Swedish or to Swedish information translated into Arabic or to information from their country of origin. It is therefore important to link future research on HL with evidence from information literacy to widen our understanding of how people seek out and use information in everyday life [50].

Another explanation for the difference in our results could be that Syrian and Iraqi migrants who have entered Europe are digitally literate [51]. Furthermore, digital communication plays a vital role in keeping migrants in contact with their relatives in their home country, with siblings who fled to other counties, and with peer refugees whom they met during their flight as well as when planning their flight [52]. That our results show that Arabic speakers’ eHL is less affected than CHL is therefore not surprising. According to this, Internet and eHealth solutions should be seen as appropriate channels for disseminating health information to Arabic-speaking migrants. Furthermore, it has been found that smartphone ownership is significantly associated with higher probabilities of belief in ability to obtain needed health information, suggesting that the use of digital technology may play a role in increasing health care access [53]. Yet, as nearly all online health information is text based, it is only available to people who are literate and able to use the internet. Migrants with limited education and low literacy may be inexperienced with the internet and are therefore not capable to seek and use digital information [54] as well as that individuals with low HL are less likely to access health information on the internet [39].

It is also known that migrants have had difficulties obtaining accurate health information during the COVID-19 pandemic [47, 50] and that the COVID-19 pandemic has been an “infodemic”, i.e. falsified information [32, 50, 55] has made it difficult to know what information can be relied on or not [50]. A sufficient level of HL not only helps individuals to evaluate the credibility of the information, but also to understand the reasons behind a recommendation and to reflect on the possible consequences of their actions [32]. Thus, HL is increasingly recognised as an important health-determining factor among migrants [56]. In Sweden, refugees were overrepresented, especially those from Somalia, Iraq, and Syria, among those who died due to COVID-19 infection [47]. Regardless of the unfortunate consequences of the COVID-19 pandemic, this could be an opportunity to assess and improve HL. The COVID-19 pandemic will not be the last crisis we face, and people and governments need to learn how to manage these crises and unexpected events. Media and information staff with the necessary knowledge about the construction and spreading of organised information can play a crucial role in coping with crises and unexpected events [57]. There is also a need to find new ways to reach migrants and refugees with culturally sensitive and understandable health information, preferably by using the Internet as a source [47]. Furthermore, the healthcare and public health sector should design tailor-made strategies that to a greater extent consider the fact that there are many people with limited HL in the population. In other words, it is important to help them to become more HL-friendly organisations [58] and responsive to HL by enhance communication procedures and guidelines, and verbal and written communication [59]. Yet, migrants should be engaged when planning, implement and evaluate these strategies through cultural mediators in health care and patients’ organizations [60].

### Study limitations

There are some limitations to be noted. The two language groups were not equal in terms of socio-demographic variables, and we therefore proceeded with the regression analysis where the differences were adjusted for. The sampling procedure was a snowball and a convenience sampling, and the study population may therefore not be a representative sample of the general Arabic and Swedish-speaking populations in Sweden. According to socio-demographic statistics regarding Arabic speaking migrants from Iraq and Syria in Sweden women and high-educated seems to be overrepresented in our study [61]. According to socio-demographic statistics regarding the Swedish speaking population there is somewhat overrepresentation of high educated participants as well as a higher mean age in our sample compared to the general Swedish population [62].

As described above, we do not know if Arabic speakers related to information in Swedish or Arabic or information from their country of origin when answering the eHEALS. Furthermore, we do not have any data as to whether the Arabic-speaking subgroup had access to a professional translator during their contacts with the healthcare services. That could influence the dimension of *understanding* in the HLS-EU-Q16 because the translator is the person mediating the information.

## Conclusions

CHL and eHL differ between native Arabic-speaking migrants and native Swedish-speaking residents, but also between Arabic speakers who have lived different lengths of time in Sweden. Though it seems that the eHealth literacy is less affected by language spoken, the Internet is suggested to be an appropriate channel for disseminating health information to Arabic-speaking migrants.

## Supplementary Information

**Additional file 1.**

## Abbreviations

CHL: Comprehensive health literacy
eHealth: Electronic health
eHEALS: eHealth literacy scale
eHL: eHealth literacy
HL: Health literacy
HLS-EU-Q16: Health literacy survey European questionnaire
