# Supplementary material for: Health literacy and e-health literacy among Arabic-speaking migrants in Sweden: a cross-sectional study
Source: BMC Public Health. 2021 Nov 25;21:2165. doi: 10.1186/s12889-021-12187-5 (PMC8614220; doi:10.1186/s12889-021-12187-5)
Supplement: Supplementary file 1 — Additional file 1. [file 12889_2021_12187_MOESM1_ESM.docx]

**Supplementary table 1.** Logistic regression predicting the probabilities of having limited HL (HLS-EU-Q16) in the Arabic speaking study population.

| Variables | Crude OR | (95% CI) | | Adjusted OR | (95% CI) | |
| --- | --- | --- | --- | --- | --- | --- |
| Biological sex |  |  |  |  |  |  |
| Male | Ref. |  |  | Ref. |  |  |
| Female | 1.91** | 1.20 | 3.06 | 1.68 | 0.90 | 3.14 |
| Age |  |  |  |  |  |  |
| 19–24 | Ref. |  |  | Ref. |  |  |
| 25–54 | 0.83 | 0.24 | 2.83 | 1.26 | 0.21 | 7.52 |
| 55–64 | 0.65 | 0.17 | 2.51 | 1.00 | 0.14 | 7.28 |
| 65+ | 0.44 | 0.10 | 2.06 | 0.72 | 0.07 | 7.19 |
| Education |  |  |  |  |  |  |
| Academic education | Ref. |  |  | Ref. |  |  |
| 7–12 years | 2.36** | 1.43 | 3.88 | 1.76 | 0.92 | 3.39 |
| 0–6 years | 2.93* | 1.19 | 7.22 | 2.87 | 0.57 | 14.52 |
| Length of stay in Sweden (years) | 0.94** | 0.91 | 0.97 | 0.94** | 0.91 | 0.98 |
| General self-perceived  health |  |  |  |  |  |  |
| Very good or good | Ref. |  |  | Ref. |  |  |
| Neither, bad, or very bad | 1.82 | 0.74 | 4.46 | 0.92 | 0.32 | 2.69 |
| Frequency of Internet use |  |  |  |  |  |  |
| Every or several days per  week | Ref. |  |  | Ref. |  |  |
| One day per week or less | 4.39* | 1.27 | 15.16 | 2.27 | 0.49 | 10.46 |
| Usability of the Internet |  |  |  |  |  |  |
| Useful or very useful | Ref. |  |  | Ref. |  |  |
| Unsure | 2.32** | 1.29 | 4.18 | 1.78 | 0.69 | 4.65 |
| Not very useful or  not useful at all | 3.39* | 1.33 | 8.62 | 0.84 | 0.22 | 3.23 |
| Importance of the Internet |  |  |  |  |  |  |
| Important or very  important | Ref. |  |  | Ref. |  |  |
| Unsure | 3.23** | 1.72 | 6.07 | 2.20 | 0.84 | 5.81 |
| Not very important or  not important at all | 4.75** | 1.76 | 12.82 | 8.04* | 1.51 | 42.94 |
| Ref. = reference category Adjusted OR (full model) R^2^ = 6.08 (Hosmer & Lemeshow), 0.19 (Cox & Snell), 0.26 (Nagelkerke). Model ꭓ^2^ (1) = 47.4, p < 0.001. *Significant at p < 0.05 **Significant at p < 0.001. Abbreviations: CI: Confidence interval; OR: Odds ratio | | | | | | |
